# Is congenital anosmia protective for Parkinson’s disease triggered by pathogenic entrance through the nose?

**DOI:** 10.1038/s41531-022-00425-5

**Published:** 2022-11-10

**Authors:** Artin Arshamian, Behzad Iravani, Johan N. Lundström

**Affiliations:** 1 Department of Clinical Neuroscience, Karolinska Institutet, Stockholm, Sweden; 2 Department of Neurology and Neurological Science, Stanford University, Stanford, USA; 3 Monell Chemical Senses Center, Philadelphia, PA USA

**Keywords:** Olfactory system, Parkinson's disease

## Abstract

The prevalence of smell loss in Parkinson’s Disease (PD) patients greatly exceeds that of the characteristic motor symptoms defining the disease by several years. One hypothesis of the cause of PD states that it is initiated in the olfactory bulb — the critical first central processing stage of the olfactory system — and that the olfactory nerve might serve as an entry point to the OB for pathogens or environmental components. But what if there was no OB to start with? Recent data demonstrate that cortical, but not peripheral, blindness acts as a protective factor against schizophrenia and other psychotic disorders. We hypothesize that individuals with the rare diagnose Isolated Congential Anosmia (ICA) are immune to PD given that they are born without OBs. If true, it would strongly support the theory that PD might start in the bulb. However, if one could identify even one single PD patient with an established ICA diagnosis with non-existing OBs, a so-called black swan, this would effectively falsify the hypothesis. In this commentary, we model the likely occurrence of such potential comorbidity and we postulate that it is possible to find this black swan; a finding that would falsify a salient hypothesis within the PD research community.

It has been suggested that congenital blindness could be protective against schizophrenia and other psychotic disorders where the resulting cortical reorganization might prevent disturbances of the eye later in life^1^, a known risk factor for schizophrenia^2^. We postulate that another rare congenital sensory disorder may protect from a central disorder, in this case Parkinson’s disease (PD). In PD, the reported prevalence of smell loss varies but larger studies using objective sensory tests commonly report the prevalence as 90–96%^3,4^, thereby exceeded the prevalence of the characteristic motor symptoms defining the disease and a clear olfactory loss often occurs years prior to most other prodromal symptoms^5^. One of several hypotheses of the cause of PD states that within the brain, the disease is initiated in the olfactory bulb (OB)^6^ the critical first central stage of the olfactory system and only entry point for olfactory signals to the brain. It is not known why the OB is an early area of insult in PD but it has been proposed that the olfactory nerve serves as an entry point for pathogens or environmental components, through which they gain OB access through the blood brain barrier^7^. Indeed, the olfactory nerve is known to transport such heavy particles as soluble metals^8^ and this mechanism is, among others, a known entryway to the OB for the SARS-CoV-2 virus^9^. Once there, it has been proposed that the pathogens trigger intraneuronal Lewy Bodies (LB) which aggregate and spread from the OB and subsequently cause the progression of PD’s pathology^6^. But what if there was no OB to start with and therefore no entryway for pathogens or environmental components to use the olfactory nerve to gain entry to the brain?

Isolated congenital anosmia (ICA) is a rare condition where otherwise healthy individuals are born without a sense of smell and with mostly aplastic (on rare occasions, hypoplastic) OBs^10,11^. In addition, absence of OBs is associated with degeneration of the olfactory nerve as well as most olfactory neurons in the olfactory epithelium^12^, thereby excluding access via the olfactory system at multiple stages. Here we propose a novel hypothesis that individuals with ICA might be immune to PD given the absence of their OB. If true, it would strongly support the notion that this type of PD starts in the bulb. However, the opposite could serve as a so-called black swan — an event which seems impossible, but which has very far-reaching consequences. If one could identify even one single PD patient outside the atypical parkinsonism subtypes with an established ICA diagnosis and aplastic OBs, this would effectively falsify this theory of how PD is initiated.

What would be the chance of finding such a patient? Using North America as an example given the available data, combining the USA demographics and the North American PD prevalence^13^ (Fig. 1a, b) with the prevalence of ICA (1/10,000)^14,15^, we calculate that while the probability is low, it is a very much possible question to settle (Fig. 1c). For example, the probability of finding a 65-year-old individual in the general US population with both ICA and PD can be estimated to 0.18‰. While admittedly difficult to find, a multitude of rare diseases exists that display lower prevalence numbers^16^.

**Fig. 1:**
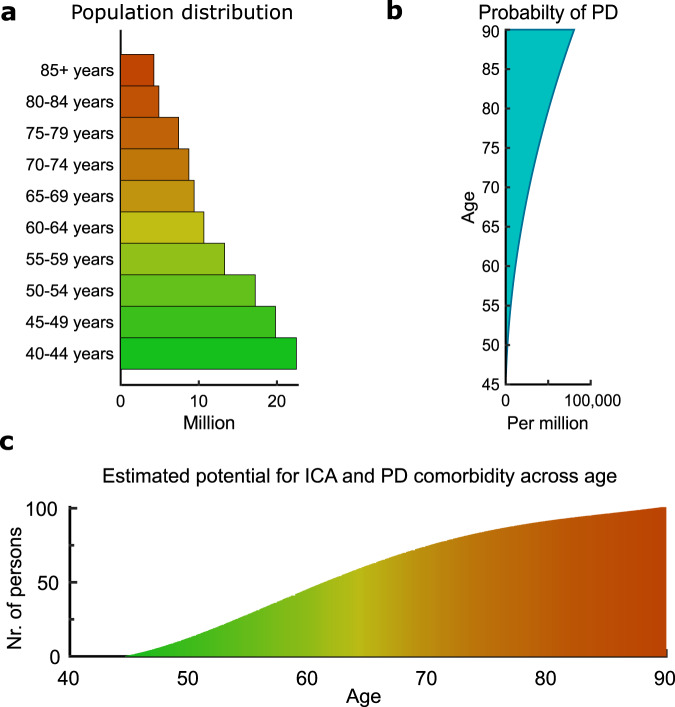
Number of potential individuals with both ICA and PD as a function of age in the US. **a** Demographic of age distribution in the US (year 2000). **b** Probability of Parkinson’s disease (PD) in North America as a function of age. **c** Estimated number of potential individuals with both isolated congenital anosmia (ICA) and PD in the US general population according to age (2018).

Finding this black swan would only serve to falsify the specific hypothesis that the OB serves as an initial staging area through which the disease propagates. It does not falsify the PD diagnose in general given that, for example, atypical parkinsonism subtypes do not display olfactory dysfunctions^17^. However, a critical notion is that because the OB is involved in PD progression in multiple ways, identifying and studying these individuals would in addition provide the community with unique insights into the OBs role in PD progression. In conclusion, although these individuals, if they exist, would be hard to find, we urge clinicians to assess their records for PD patients with both an ICA diagnosis and aplastic OBs. Finding this black swan would falsify a salient hypothesis within the PD research community and open up new research avenues.

## Reporting summary

Further information on research design is available in the Nature Research Reporting Summary linked to this article.

## Supplementary information

Reporting Summary

## Data Availability

US demographic values was obtained from open databases with references provided within the manuscript.

## Code availability

Codes to generate figures can be obtained with contact with communicating authors.
